# The prevalence of SARS-CoV-2 antibodies in triage-negative patients and staff of a fertility setting from lockdown release throughout 2020

**DOI:** 10.1093/hropen/hoab028

**Published:** 2021-07-27

**Authors:** Corina Manolea, Andrei Capitanescu, Roxana Borș, Ioana Rugescu, Melihan Bechir, Claudia Mehedintu, Valentin Varlas

**Affiliations:** 1 Department of Obstetrics and Gynecology, ‘Carol Davila’ University of Medicine and Pharmacy, Bucharest, Romania; 2 Department of Assisted Reproduction, Columna Medical Center, Bucharest, Romania; 3 Hemodialysis Unit, ‘Marie Curie’ Pediatric Clinical Emergency Hospital, Bucharest, Romania; 4 Department of Obstetrics and Gynaecology, Filantropia Clinical Hospital, Bucharest, Romania; 5 Department of Cells, National Transplant Agency, Bucharest, Romania; 6 Dept of Obstetrics and Gynecology, Infertility Center, Regina Maria Medical Network, Bucharest, Romania; 7 Department of Obstetrics and Gynecology, Nicolae Malaxa Clinical Hospital, Bucharest, Romania

**Keywords:** severe acute respiratory syndrome coronavirus 2, SARS-CoV-2 antibodies, assisted reproduction, ART, serological testing, IgG seroprevalence, SARS-CoV-2 prevalence trends, coronavirus disease 2019

## Abstract

**STUDY QUESTION:**

What is the prevalence of severe acute respiratory syndrome coronavirus 2 (SARS-CoV-2) antibodies in triage-negative patients undergoing ART and fertility care providers after lockdown release and throughout 2020?

**SUMMARY ANSWER:**

Out of the triage-negative patients whose blood samples were assessed for SARS-CoV-2 antibodies over 6 months, 5.2% yielded positive results with a significantly higher rate in health care workers (HCWs) and a significant month-by-month increase in those with evidence of antibodies.

**WHAT IS KNOWN ALREADY:**

Patients of reproductive age are more prone to asymptomatic or minimal forms of coronavirus disease 2019 (COVID-19) as compared to older age groups, and the identification of those with active infection and those already exposed (and probably immunized) is important for safety and cost-effective use of testing resources in the fertility setting. Data on the prevalence of SARS-CoV-2 in ART patients are limited and encompass short time frames; current rates are unknown. There is also no consensus on the optimal way of screening triage-negative ART patients in moderate/high-risk areas.

**STUDY DESIGN, SIZE, DURATION:**

A prospective longitudinal unicentric study on triage negative ART patients (n = 516) and clinical staff (n = 30) was carried out. We analyzed 705 serological tests for SARS-CoV-2 sampled between 17 May 2020 (the first working day after lockdown release) up to 1 December 2020, to assess the positivity rates for SARS-CoV-2 antibodies.

**PARTICIPANTS/MATERIALS, SETTING, METHODS:**

We collected data on the serological status for IgM and IgG antibodies against SARS-CoV-2 in 516 triage-negative men (n = 123) and women (n = 393) undergoing ART at a private fertility center and 30 HCWs that were at work during the study period. Antibodies were detected with a capture chemiluminescence assay (CLIA) targeting the highly Immunogenic S1 and S2 domains on the virus spike protein. We also analyzed the molecular test results of the cases exhibiting a positive serology.

**MAIN RESULTS AND THE ROLE OF CHANCE:**

The data showed that 5.2% of the triage-negative ART patients had a positive serological result for SARS-CoV-2, with an overall conversion rate of 2.1% for IgG and 4.6% for IgM. There was no significant difference in seroprevalence between sexes. The small cohort (n = 30) of HCWs had a markedly increased seroprevalence (12.9% for Ig M and 22.6% for IgG). The highest seropositivity in our cohort was recorded in November (16.2%). The IgM positivity rates revealed significant monthly increments, paralleling official prevalence rates based on nasopharyngeal swabs. No positive molecular tests were identified in cases exhibiting a solitary positive IgG result. We show that despite a 6-fold increase in the number of ART patients with a positive serology between May and December 2020, most of our patients remain unexposed to the virus. The study was undertaken in a high-risk area for COVID-19, with a 20-times increase in the active cases across the study period.

**LIMITATIONS, REASONS FOR CAUTION:**

The geographical restriction, alongside the lack of running a second, differently-targeted immunoassay (orthogonal testing), could limit the generalizability and translation of our results to other fertility settings or other immunoassays.

**WIDER IMPLICATIONS OF THE FINDINGS:**

The low positivity rates for IgG against the SARS-CoV-2 spike protein seen at the end of 2020 imply that most of the fertility patients are still at risk for SARS-CoV-2 infection. Until mass vaccination and other measures effectively diminish the pandemic, risk mitigation strategies must be maintained in the fertility units in the foreseeable future. Patients with a solitary IgG+ status are most likely ‘non-infectious’ and can elude further testing without giving up the strict use of universal protective measures. With increasing seroprevalences owing to infection or vaccination, and with the consecutive increase in test performance, it is possible that serological screening of ART patients might be more cost-effective than PCR testing, especially for the many patients with repeat treatments/procedures in a time-frame of months.

**STUDY FUNDING/COMPETING INTERESTS:**

This research received no external funding. All authors declare having no conflict of interest with regard to this trial.

WHAT DOES THIS MEAN FOR PATIENTS?

This study looks at how many of the individuals attending a fertility clinic during the second half of 2020 show evidence of recent or past infection with the virus causing coronavirus disease 2019 (COVID-19). Infection was evaluated in over 500 people undergoing assisted reproduction, by a blood test measuring specific antibodies (proteins made by the body in response to foreign invaders such as viruses and bacteria). All of those tested reported no COVID-19 symptoms or known exposure around the time of their fertility treatment.

We found that roughly 5% of the women and men tested returned positive antibody readings, with no significant difference between the sexes; 2% presented with antibodies suggesting past infection while twice as many had antibodies suggesting recent or present infection. Among those tested at the end of the study, one out of six were positive. Close to 2% of all the patients were detected as having an active infection and had to postpone the treatment.

This means that although the number of those with infection and immunity is increasing, the majority of the infertility patients from a high-risk area for COVID-19 remain unexposed to the virus at the end of 2020. The use of protective measures and testing for COVID-19 in the fertility clinics will most probably go on throughout all 2021.

## Introduction

Most of the year 2020 has been consumed by a viral pandemic that seems to linger on (ECDC, 2020) and to reshape healthcare worldwide.

Infertility is a pressing medical condition, time- and pandemic-sensitive (Alviggi *et al.*, 2020), which is diagnosed in about one-fifth of reproductive-aged couples, amounting to 186 million couples globally (WHO, 2020); many of those affected will succeed in their dream of parenthood with the use of ART.

One of the main concerns surrounding the generation of medically assisted pregnancies in the middle of a pandemic environment was to prevent infection in the fertility clinic and, consequently, to identify optimal screening algorithms for the detection of asymptomatic or pre-symptomatic cases of infection with severe acute respiratory syndrome coronavirus 2 (SARS-CoV-2) (ARCS and BFS, 2020; ASRM, 2020; ESHRE, 2020; Romanian MoH, 2020).

In non-emergent non-coronavirus disease 2019 (COVID-19) care, tele triage and testing are considered two indispensable steps for ensuring safety for patients and staff (AMA, 2021). Testing for viral RNA (through quantitative real-time RT-PCR) is widely accepted for confirming active infection with SARS-CoV-2 in symptomatic individuals, while serological testing is used to complement and remedy the nucleic acid amplification tests (NAAT) detection, to establish the timeline of the infection, to screen asymptomatic populations and to quantify vaccine responses (Böger *et al.*, 2020; La Marca *et al.*, 2020; Petherick, 2020; Vandenberg *et al.*, 2020; Wang *et al.*, 2020).

The rates and trends of SARS-CoV-2 prevalence in the fertility practice are unknown. Existing recommendations for SARS-CoV-2 screening in the fertility setting issued by the professional bodies insist on triage and symptom-driven testing, and are not aligned (La Marca and Nelson, 2020; Sparks and Kresowik, 2021), which is understandable considering the lack of evidence on best practice (Papathanasiou, 2020). One piece of the information upon which cost-effective screening protocols and preventative measures can be devised and optimized is knowledge on SARS-CoV-2 prevalence trends in ART patients with a negative triage questionnaire. This study aims to assess SARS-CoV-2 IgG and IgM seroprevalence in triage-negative people who are attending and working in a fertility setting throughout the second half of 2020.

## Materials and methods

### Study design and population

We performed a prospective longitudinal unicentric study with a duration of 6 months, aiming to report the serological status for IgG and IgM to SARS-CoV-2 in triage-negative infertility patients and clinical staff overtime.

All patients eligible for undergoing ART treatments in a sizable private fertility unit in Bucharest, a 2 million people COVID-19 hotspot in Romania were offered the option of serological testing during the initial stages of their treatment or molecular testing just before the fertility intervention. Seventy-three percent of all the patients consented to serological screening. They could opt out at any time, but since universal testing was employed by the clinic, a negative molecular test was required just before undergoing the intervention.

In total, 516 triage-negative patients undergoing high-complexity fertility treatments (oocyte retrieval, surgical sperm extraction, frozen embryo transfer: FET) were included.

All underwent a recorded telephonic triage questionnaire before the initial presentation using the model provided by ESHRE and were again clinically triaged by a nurse at each visit throughout their treatment.

We also investigated the antibody status of 30 patient-facing HCWs providing reproductive care in the center.

A total of 713 serological tests were sampled between the 17th May 2020 (the first working day after lockdown release) and the 1st December 2020 and analyzed for the presence of SARS-CoV-2 antibodies. A total of 125 of the individuals (17.7%) were sampled twice and 25 (3.5%) were sampled three times across the study period for repeating fertility interventions, provided their first result was negative (Table I). Eight tests reporting equivocal results were excluded from the analysis. As per local protocol, patients and HCWs that had a positive serological result underwent subsequent molecular testing no more than 48 h apart (Roche Cobas 6800 SARS-CoV-2 test, Roche, Basel, Switzerland).

**Table I:** Cohort characteristics and differences between patients and health care workers.

Parameter	Overall	Health care worker		Univariate analysis		*P* value
		Yes (n = 30)	No (n = 516 patients)	Odds ratio	95% CI	
**Total tests performed**	705	31	674	NA	–	NA
**Individuals with 2 tests**	125 (17.7%)	1	124	NA	–	NA
**Individuals with 3 tests**	25 (3.5%)	0	25	NA	–	NA
**Individuals with 4 tests**	6 (0.9%)	0	6	NA	–	NA
**Individuals with 5 tests**	3 (0.4%)	0	3	NA	–	NA
**Females (%)**	421 (77.1)	29 (93.5)	529 (78.5)	0.25	0.06–0.96	0.043
**Age (years)**	35 [32; 40]	35 [31; 42.5]	35 [32; 40]	NA	–	0.714
**Positive IgM (%)**	35 (5)	4 (12.9)	31 (4.6)	3.07	1.06–8.96	0.037
**Positive IgG (%)**	21 (3)	7 (22.6)	14 (2.1)	13.75	5.22–36.41	<0.001
**Both IgM and IgG positive (%)**	14 (2)	4 (12.9)	10 (1.5)	9.83	3.06–31.84	<0.001
**IgM or IgG positive (%)**	42 (6)	7 (22.6)	35 (5.2)	5.35	2.19–12.95	<0.001
**PCR positive among IgM+ or IgG+ (%)**	9 (21.4)	2 (28.6)	7 (20)	NA	–	0.61

Data presented as median and interquartile interval or numbers (n) and percentages (%).

Univariate analysis was performed to compare patients with HCWs.

Overall and monthly crude seroprevalences were calculated as the number of reactive cases divided by the number of cases tested. Adjusted prevalence was obtained after controlling for the test performance. The temporal changes in seroprevalence rates across the study period were also evaluated.

Additionally, we analyzed official data regarding Romania`s monthly SARS-CoV-2 general prevalence rates recorded between 25 February 2020, when the first case of infection was reported, and 25 February 2021 (www.worldometers.info, 2021) and performed ANOVA between official rates and our monthly prevalence rates in the ART population.

We extrapolated data and adjusted for our cohort for a 3-month follow-up, estimating SARS-CoV-2 rates in the ART population by late February 2021, 1 year after the first case was confirmed in Romania.

### Serological tests for SARS-CoV-2

The blood samples were obtained voluntarily through venipuncture during one of the monitoring visits to the clinic. Sampling occurred no later than 3 days before the fertility intervention. The serological results were received in a few hours. All the patients were informed and provided consent.

HCWs that were at work (hence, temperature check and triage-negative) during the study period were also tested voluntarily, only once except for one who was tested twice.

IgG and IgM antibodies targeting the S1 and the S2 domains of SARS-CoV-2 spike protein were tested in serum samples using a commercially available capture chemiluminescence immunoassay (CLIA) kit (LIAISON^®^ SARS-CoV-2 S1/S2 IgG and LIAISON^®^ SARS-CoV-2 IgM, DiaSorin, Sallugia, Italy).

The CLIA technique is similar to the more popular ELISA except for the specific enzyme-catalyzed substrate used, which is luminol, translating into a change in luminescence (not color) in the CLIA assays. CLIA is fully automated and seems to exhibit even higher sensitivities than ELISA (Wang *et al.*, 2020).

The DiaSorin S1/S2 IgG is a quantitative test for determining the IgG antibodies directed at two subunits of the spike protein involved in binding (S1) and fusion (S2) of SARS-CoV-2 to cells. In individuals with normal or compromised immunity, the assays targeting the highly immunogenic sites of the spike protein are more sensitive than the ones targeting other viral antigens (Prendecki *et al.*, 2020). Given the correlation with neutralizing activities, spike antibodies are also relevant for functional immunity (Premkumar *et al.*, 2020; Wajnberg *et al.*, 2020).

Briefly, magnetic beads are coated with both S1 and S2 antigens to which specific antibodies attach (solid phase). Complexes of mouse monoclonal antibodies to human IgG and isoluminol are created and put in contact with the solid phase. The complexes bind to the SARS-CoV-2 antibodies present in the sample, producing a light signal that is read by a photomultiplier.

Samples with signal levels above the manufacturer`s cutoff of ≥ 15 arbitrary units (AU)/ml were defined as positive, and samples below 12 AU/ml were defined as negative. Results between 12 and 15 AU/ml are reported as equivocal, and re-testing is advised (www.diasorin.com). We excluded equivocal results from our analysis.

According to the manufacturer, the S1/S2 IgG assay has a clinical sensitivity of 90.7% for samples tested 5–15 days after infection and 97.9% for later than 15 days; the specificity is 98.6%.

IgM against the receptor binding domain of SARS-CoV-2 was evaluated qualitatively by the same approach, with results interpreted as positive or negative against an index of 1.1 described by the manufacturer. The clinical sensitivity for the IgM assay used in this study is 91.5% for Days 8–14, and 94% for Days 15–30 post-infection; the specificity is 99.3%. The combined assays offer a clinical sensitivity that reaches 98.3% when testing is undertaken after Day 15 postinfection.

External validation studies of the assay used in this work provided ‘real-world’ analytical and clinical performances of the DiaSorin assay that were close to the ones reported by the manufacturer, with the time-dependent increase of accuracy inherent to the serological assays. The populations used for external validation comprise adults over 18 years of age with a previous positive PCR test for SARS-CoV-2 and different forms of infection ranging from asymptomatic to critical, with no evidence of a difference in immunoassay sensitivity by infection severity (Public Health England, 2020; The National SARS-CoV-2 Serology Assay Evaluation Group, 2020; Tré-Hardy *et al.*, 2020; Turbett *et al.*, 2020).

### Ethical considerations

No personal data that could identify any person was included. Approval from the Ethical Committee of Columna Medical Center (reference: CMC-1330-15052020) was obtained before the initiation of sampling and data collection.

### Statistical analysis

We performed statistical analysis and graphs using Analyze IT 5.5 (Microsoft Office Excel Add-on, Leeds, UK). The data had a non-Gaussian distribution and were presented as the median and the interval between the quartiles. The differences in quantitative parameters were tested using nonparametric tests. Qualitative data were compared with the Chi-square test. We considered statistical significance at a *P*-value lower than 0.05.

## Results

A total of 713 blood tests for SARS-CoV-2 were analyzed and 98.8% of them produced unequivocal results. The median age of the individuals included in the study (516 triage-negative patients and 30 HCWs was 35 years, interquartile range [32; 40]) . Three quarters of them (77.1%) were women. After consenting, no patient opted out of serological testing.

Out of the 705 blood samples that produced valid readings for SARS-CoV-2 antibodies, 42 yielded positive results, giving a raw seroprevalence of 6%. After adjusting for test performance, the seroprevalence across the study period was 6.93%.

The overall seroconversion rate was 3% for IgG and 5% for IgM, with high variability in the antibody titers and a non-significant difference between sexes (*P* value: 0.13). Two percent of the samples were reactive for both antibodies.

In the patient group, 2.1% (n = 21) had evidence of IgG antibodies against SARS-CoV-2 and 4.6% (n = 35) had positive IgM results.

Regarding the HCW group, the prevalence of antibodies was 12.9% for Ig M and 22.6% for IgG, markedly increased as compared to the patient population (Table I).

The reader must be aware that more than half of the HCWs were tested in the first 2 months after recommencing clinical activities (when national data on HCWs revealed a high prevalence rate); half of the remaining were tested in November, during a pandemic peak (Supplementary Table SI).

The first cases of patients and staff with positive IgG results were seen in May, immediately after lockdown release (1% seroprevalence) with significantly higher numbers thereafter (6% during autumn).

**Figure 1.**
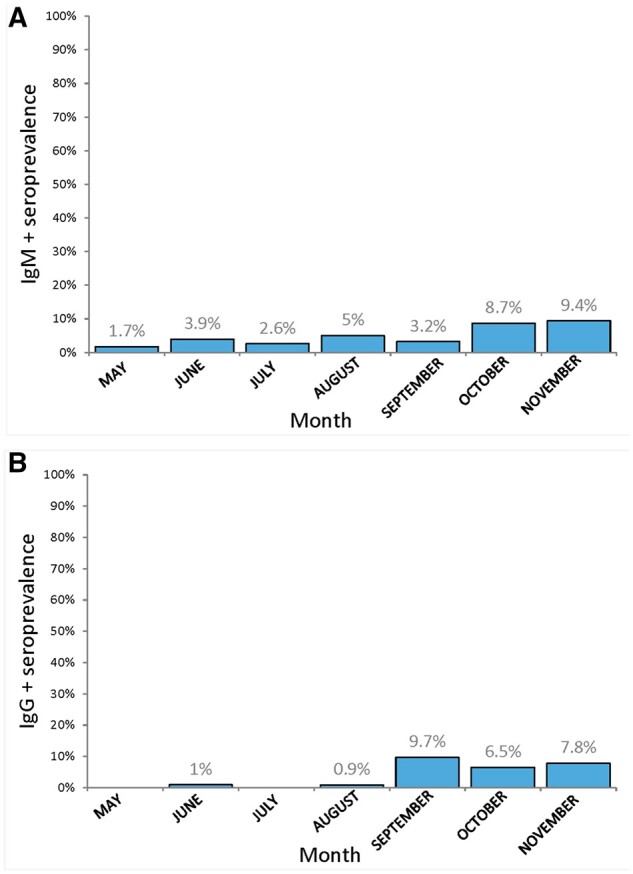
**Monthly seroprevalence of SARS-CoV-2 antibodies in 516 triage-negative ART patients from lockdown release (15^th^ of May) in Bucharest, Romania, throughout 2020**. SARS-CoV-2: severe acute respiratory syndrome coronavirus 2.

Monthly antibodies seroprevalences in the patient group rose gradually after lockdown release: from 1.7% in the second half of May (n = 58), to 5.5% in August (n = 220), to 10.9% in November (n = 64) (Fig. 1).

When analyzing the reactivity rates for IgG and IgM recorded monthly, the difference between the time intervals was found to be significant (*P* < 0.01) (Supplementary Table SI).

The number of individuals exhibiting a positive serological result for one or both antibodies increased 6-fold during the study period.

We analyzed official data regarding national monthly SARS-CoV-2 general prevalence rates recorded between 25 February 2020, when the first case of infection was reported, and 25 February 2021 (www.worldometers.info, 2021). We performed ANOVA between the official prevalence rates and our data and found a correlation of statistical significance (*P*-value 0.0158) with an Odds Ratio of 0.512 (95% CI: 0.145–0.878) (Supplementary Table SII).

**Figure 2.**
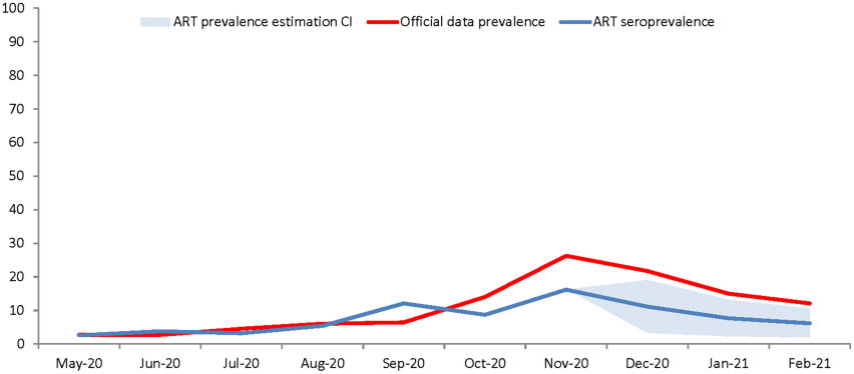
**Prevalence dynamics in the general population and the ART population (May 2020–February 2021).** Prevalence in the general Romania population is based on RT-PCR national data. Prevalence in the ART population is based on monthly positivity rates of IgM or/and IgG.

We extracted data and adjusted our cohort for a 3-month follow-up until the date of submission, estimating a plateau followed by a decrease in the ART population testing positive for antibodies by the end of February 2021, alongside the decrease in cases officially recorded in the national population during this period (Fig. 2).

**Figure 3.**
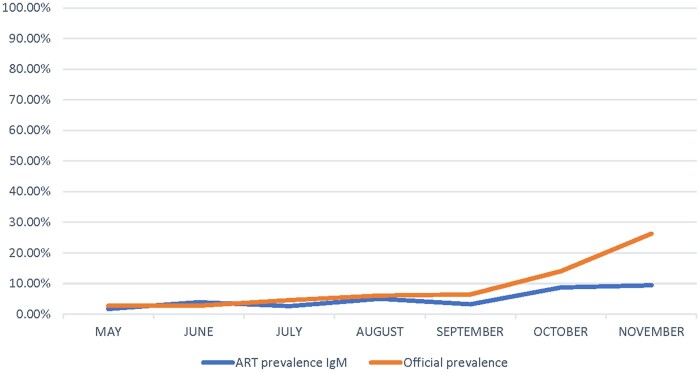
**Monthly IgM prevalence rates in the ART patients versus national monthly prevalences based on nasopharyngeal swabs (May–December 2020).** Data presented as %.

When evaluating the trend in IgM positivity, we found the monthly increase in IgM positivity rates to be significant and to parallel the increase in national PCR-based prevalence rate (Fig. 3 and Supplementary Table SIII).

Although outside the scope of this antibody prevalence study, we analyzed the PCR results in all individuals that tested positive for either IgM or IgG. Out of the 42 PCR tests carried out in patients with a positive IgM or/and IgG serology, 9 (21.4%) were positive. Only 34.61% of the patients with IgM+ results had a corresponding positive PCR, while no positive molecular tests were seen among the patients that were positive exclusively for IgG (Supplementary Table SIV).

Close to 2% of the individuals in our cohort had a positive result for both serology and PCR.

Concerning the form of infection, and based on the recollection of COVID-19 symptoms, 65% of those with positive serology were asymptomatic, 25% reported minimal symptoms, and none required hospital care.

## Discussion

In our cohort of over 500 patients admitted for ART with a triage-negative result between May and December 2020, we found a low overall conversion rate for SARS-CoV-2 antibodies (5.2%), meaning that current patients remain largely unexposed to the virus. Still, prevalence rates reveal a sustained monthly increase after the national lockdown was lifted on 15 May 2020, surging to 10.9% in November.

The temporal evolution of IgM and IgG prevalence in the study population closely followed the local pandemic pattern: SARS-CoV-2 diffusion increased with relaunching free movement and tourism during the summer months, and the highest rate of seropositivity in our study was recorded during late autumn, coincident with the expansion of the second pandemic wave in Romania and many European countries (ECDC, 2020).

The seroprevalence of IgG antibodies in ART patients recorded here (2.1%) is consistent with the locally reported prevalence of 2.8% of SARS-CoV-2 IgG in 1831 healthy Romanian blood donors of reproductive age sampled throughout an increase in cases (July–September 2020) (Olariu *et al.*, 2021) .

Despite the overwhelming influence of SARS-CoV-2 infection on society and healthcare, many serosurveys reveal rather low positivity figures, even in hotspots of COVID-19. Data from Wuhan, Italy, Switzerland, and Spain show serological conversion rates of 3.6–10.8%, recorded weeks apart from the peak of infection (Eckerle and Meyer, 2020). These studies were carried out in the general population and, hence, in low pretest probability settings, similar to ours.

In line with our results, others show moderate but increasing seroprevalences over time. A serosurvey undertaken 1 week after the summer pandemic peak in the Geneva area revealed an increase in IgG seroprevalence of 6 % points over the course of 5 weeks (from about 5% to about 11%) (Stringhini *et al.*, 2020). A study investigating close to 1 million blood donor samples in the USA found an overall 2.99% prevalence of SARS-CoV-2 IgG in first-time donors and a doubling in reactivity across summer months (Dodd *et al.*, 2020).

Information on the prevalence of SARS-CoV-2 in ‘real-world’ fertility practice is scarce and limited by a short time frame of the sampling period.

Data from Spain report seroconversion rates of 3.6% and 0.7% for IgG and IgM, respectively, in a population of over 1500 triage-negative women undergoing ART in 17 clinics, sampled in May 2020 (González-Ravina *et al.*, 2020). These figures are higher than our May results but can be explained by the country-specific temporal evolution of the pandemic, with Spain being one of the earliest most affected countries in Europe (ECDC, 2021).

There is no agreement over COVID-19 testing in the ART clinic: which type of test is the most cost-effective, when to apply it, and whether to go for universal testing (ASRM, 2020; ESHRE, 2020). In real-life clinical practice, triage-negative ART patients are usually screened with a PCR test once or even twice (Joint IFS-ISAR-ACE, 2020) throughout their treatment, with the inherent costs and risk of false-negative results, especially in those with no or minimal symptoms (Watson *et al.*, 2020). International data reveal a very low rate of positive PCR results in triage-negative IVF patients: 0.5% in 4259 asymptomatic women undergoing IVF in Israel; no positive PCR in a cohort of 151 ART patients from New York, NY, USA; 0.8% out of 263 IVF cycles revealing a new asymptomatic infection in May–July 2020 in another New York-based fertility center (Gingold *et al.*, 2021; Robles *et al.*, 2020; Seidman *et al.*, 2020) .

In the afore-mentioned Spanish study, out of the women selected to proceed with treatment based on the serological results (IgG+/IgM- or IgG-/IgM-), only one (0.06%) had a positive PCR result when tested just before oocyte retrieval. We performed immediate PCR testing also in patients with IgM+ results and found that just one-third (34.61%) had a corresponding positive PCR and had to postpone treatment. It seems that IgM sampling may decrease cycle cancellation (González-Ravina *et al.*, 2020), as opposed to universal screening with RT-PCR just before oocyte retrieval/FET; it may also stratify as new versus previous infection, optimizing treatment scheduling. Similarly, we did not identify any positive molecular test among the patients with a solitary positive IgG. It seems that a positive IgG result might be regarded as a marker of ‘non-infectiousness’ (Denning and Kilcoyne, 2020) and can avoid further PCR testing. Still, the IgG+ status does not preclude the strict use of protective measures at least until clarification on adaptive and vaccinal immunity, reinfection, and transmissibility by the immunized (Saad-Roy *et al.*, 2020).

In the subgroup of HCWs, the high prevalence of SARS-CoV-2 antibodies must be judged keeping in mind the small sample size and the locally reported high infection rates (up to 15%) among HCWs across the specific study months when most of the fertility care providers were sampled (INSP Romania, 2020).

Studies addressing seroconversion and asymptomatic infection in HCWs that were at work in large hospitals in the UK (Shields *et al.*, 2020) and China (Zhao *et al.*, 2020) have confirmed that molecular testing consistently underestimates true infection rates (2.39% prevalence of PCR-detected infection and an overall seroconversion rate of 24.4% in the UK). One study analyzed the paired molecular and serological tests in 554 HCWs from 31 fertility and ultrasound clinics across four European countries at the time of lockdown release in May 2020. They reported a 0.19% positivity rate for NAAT testing and an overall antibody prevalence of 4.15% (Nelson *et al.*, 2020); it may be presumed that contemporary rates are significantly higher.

Two-thirds of our subjects with a positive serology did not recollect any COVID-19-specific symptoms. It is established that younger age and female sex correlate strongly with asymptomatic and mild infections (Long *et al.*, 2020; Nikolai *et al.*, 2020; Peckham *et al.*, 2020; Pollock and Lancaster, 2020; Yang *et al.*, 2020), so that ART patients are more prone to be hidden drivers of the pandemic. The accuracy and usefulness of serological assays in such individuals were questioned. Using multiple differently targeted immunoassays, it was demonstrated that most asymptomatic and mildly symptomatic individuals will seroconvert, exhibiting lower antibody titers than the severely affected, but with detectable levels lasting for at least several months (Choe *et al.*, 2021; Dan *et al.*, 2020) .

We admit that the geographical restriction, the monocentric nature of the study, and the small cohort of HCWs are major limitations, potentially rendering a difficult translation to other fertility settings.

Also, we did not validate our positive serological results through a second, differently targeted assay; it was shown that an orthogonal testing algorithm could identify potentially false-positive SARS-CoV-2 IgG serology results, particularly in populations with a low disease prevalence (Ripperger *et al.*, 2020).

We underline as study strengths the sampling of a focused population, using a CLIA assay with a highly immunogenic target, over a period of 6 months, illustrating the distribution of SARS-CoV-2 exposed versus naive individuals in a standalone fertility unit during the second half of 2020.

The key finding is that, despite sampling reproductive-aged, active people in a fertility setting from a high SARS-CoV-2 circulation area over 6 months, the proportion of seroconverters remains rather modest by the end of 2020. A small number of the patients attending the fertility service have positive serological and molecular results (1.74% in our cohort) and risk going undetected unless proper testing is applied. As the proportion of those with evidence of antibodies is gradually rising in our study and in other works (Bajema *et al.*, 2020; Dodd *et al.*, 2020; Stringhini *et al.*, 2020), the screening of ART patients may soon rely less on molecular tests and more on serology.

Based on the prevalence recorded in our study, we must emphasize the risks of lowering the standards of protection and screening for patients and staff. Most of them are susceptible to the virus and must be shielded even more so as vaccination programs have started to roll out worldwide (ASRM, 2021); it is also possible that vaccine hesitancy will play a role in the ART population (Dror *et al.*, 2020). Risk mitigation strategies in the fertility units must be held in place in the foreseeable future (ESHRE, 2021), alongside constant surveillance of the pandemic and of the growing body of literature in the field (Gleicher, 2020).

## Supplementary data

Supplementary data are available at *Human Reproduction Open* online.

## Data availability

The data underlying this study are available on request from the corresponding author. 

## Authors’ roles

All authors contributed to acquisition, analysis, and data interpretation, critically revised the manuscript, and gave final approval. C.M. and A.C. were also involved in conception and design. C.M. drafted the manuscript.

## Funding

This research received no external funding. 

## Conflict of interest

The authors declare no conflict of interest.

## Supplementary Material

hoab028_Supplementary_Data
