# Supplementary material for: The prevalence of SARS-CoV-2 antibodies in triage-negative patients and staff of a fertility setting from lockdown release throughout 2020
Source: Hum Reprod Open. 2021 Jul 27;2021(3):hoab028. doi: 10.1093/hropen/hoab028 (PMC8313405; doi:10.1093/hropen/hoab028)
Supplement: hoab028_Supplementary_Data [file hoab028_supplementary_data.zip › Supplementary-Table-SIV final.docx]

**Supplementary Table SIV** Negative predictive value of IgM for RT-PCR testing.

| **IgM** | **RT-PCR** | | **Total** |
| --- | --- | --- | --- |
|  | **Positive** | **Negative** |  |
| **Positive** | 7 | 24 | 4 |
| **Negative** | 0 | 4 | 31 |
| **Total** | 7 | 28 | 35 |

Only 34.61% of the patients with IgM+ results had a corresponding positive PCR, while no positive molecular tests were seen among the patients that were positive exclusively for IgG.

Data presented as numbers (n)
