# Supplementary material for: The prevalence of SARS-CoV-2 antibodies in triage-negative patients and staff of a fertility setting from lockdown release throughout 2020
Source: Hum Reprod Open. 2021 Jul 27;2021(3):hoab028. doi: 10.1093/hropen/hoab028 (PMC8313405; doi:10.1093/hropen/hoab028)
Supplement: hoab028_Supplementary_Data [file hoab028_supplementary_data.zip › Supplementary-Table-SI final.docx]

**Supplementary Table SI** Monthly serological tests for SARS-CoV-2 between May - December 2020.

|  |  | **Month** | **May** | **Jun** | **Jul** | **Aug** | **Sep** | **Oct** | **Nov** | **Total** |
| --- | --- | --- | --- | --- | --- | --- | --- | --- | --- | --- |
| **Patients** | **IgG** | negative | 58 | 101 | 153 | 218 | 28 | 43 | 59 | 660 |
|  |  | positive | 0 | 1 | 0 | 2 | 3 | 3 | 5 | 14 |
|  | **IgM** | negative | 57 | 98 | 149 | 209 | 30 | 42 | 58 | 643 |
|  |  | positive | 1 | 4 | 4 | 11 | 1 | 4 | 6 | 31 |
| **Health care workers** | **IgG** | negative | 18 | 3 | 0 | 2 | 1 | 0 | 0 | 24 |
|  |  | positive | 1 | 0 | 1 | 0 | 1 | 0 | 4 | 7 |
|  | **IgM** | negative | 19 | 3 | 1 | 2 | 1 | 0 | 1 | 27 |
|  |  | positive | 0 | 0 | 0 | 0 | 1 | 0 | 3 | 4 |
|  |  | Total tests | 77 | 105 | 154 | 222 | 33 | 46 | 68 | 705 |

SARS-CoV-2: severe acute respiratory syndrome coronavirus 2

Data presented as numbers (n)
