# Supplementary material for: The prevalence of SARS-CoV-2 antibodies in triage-negative patients and staff of a fertility setting from lockdown release throughout 2020
Source: Hum Reprod Open. 2021 Jul 27;2021(3):hoab028. doi: 10.1093/hropen/hoab028 (PMC8313405; doi:10.1093/hropen/hoab028)
Supplement: hoab028_Supplementary_Data [file hoab028_supplementary_data.zip › Supplementary-Table-SII final.docx]

**Supplementary Table SII** Analysis of variance between monthly mean prevalences in Romania (based on nasopharyngeal swabs), named “official prevalence” and in the ART population (based on the positivity of IgM and IgG antibodies).

| **Parameter** | **Estimate** | **95% CI** | |
| --- | --- | --- | --- |
| **Official prevalence** | 0.5116 | 0.1450 | to 0.8781 |

P value 0.0158

|  | May-20 | Jun-20 | Jul-20 | Aug-20 | Sep-20 | Oct-20 | Nov-20 |
| --- | --- | --- | --- | --- | --- | --- | --- |
| **ART prevalence** | 2.6 | 3.8 | 3.2 | 5.4 | 12.1 | 8.7 | 16.2 |
| **Official prevalence** | 2.71 | 2.74 | 4.56 | 6.07 | 6.42 | 14.03 | 26.27 |

Prevalence rates are presented as %.
