# Supplementary material for: The prevalence of SARS-CoV-2 antibodies in triage-negative patients and staff of a fertility setting from lockdown release throughout 2020
Source: Hum Reprod Open. 2021 Jul 27;2021(3):hoab028. doi: 10.1093/hropen/hoab028 (PMC8313405; doi:10.1093/hropen/hoab028)
Supplement: hoab028_Supplementary_Data [file hoab028_supplementary_data.zip › Supplementary-Table-SIII final.docx]

**Supplementary Table SIII** Monthly national prevalence rates (based on nasopharyngeal swabs) versus monthly IgM prevalence rates in the ART population.

.Data presented as percentages (%).

| **Month** | May | June | July | August | September | October | November |
| --- | --- | --- | --- | --- | --- | --- | --- |
| **ART patients IgM prevalence** | 1.70 | 3.90 | 2.60 | 5 | 3.20 | 8.70 | 9.40 |
| **National RT-PCR-based prevalence** | 2.71 | 2.74 | 4.56 | 6.07 | 6.42 | 14.03 | 26.27 |
